# Massive hemoptysis in pregnancy due to invasive pulmonary aspergillosis with pulmonary tuberculosis co‐infection

**DOI:** 10.1002/rcr2.1315

**Published:** 2024-03-07

**Authors:** Nurul Aisyah Abd Rahman, Boon Hau Ng, Nik Nuratiqah Nik Abeed, Muhammad Ishamuddin Ismail, Mohamed Faisal Abdul Hamid, Andrea Ban Yu‐Lin

**Affiliations:** ^1^ Respiratory Unit, Department of Medicine, Faculty of Medicine Universiti Kebangsaan Malaysia, Hospital Canselor Tuanku Muhriz Kuala Lumpur Malaysia; ^2^ Cardiothoracic Unit, Department of Surgery, Faculty of Medicine Universiti Kebangsaan Malaysia, Hospital Canselor Tuanku Muhriz Kuala Lumpur Malaysia

**Keywords:** aspergillosis, co‐infection, hemoptysis, pregnancy, tuberculosis

## Abstract

A 37‐year‐old woman, 25 weeks pregnant, experienced sudden massive hemoptysis. She had a background history of systemic lupus erythematosus (SLE) and past pulmonary tuberculosis (PTB). Emergency intubation was necessary, and bronchoscopy revealed blood pooling in both main bronchi, with active bleeding from the right upper lobe bronchus. Urgent computed tomography (CT) angiography of the bronchial artery identified a bleeding source and was successfully embolized. Antifungal and anti‐tuberculous therapy was initiated based on bronchoalveolar lavage results. Despite initial improvement, hemoptysis recurred after the third week, leading to repeat embolization, followed by a caesarean section and right upper lobectomy. Both mother and baby survived, remaining well at a 6‐week follow‐up, emphasizing the complexities of managing recurrent hemoptysis during pregnancy and potential drug interactions.

## Graphical abstract

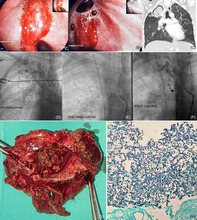

## INTRODUCTION

Post‐tuberculosis lung disease reduces life expectancy and elevates the likelihood of recurring tuberculosis infection or aspergillosis.^1^, ^2^ Aspergillosis can manifest within the cavities formed by tuberculosis, exacerbating the lesions and potentially leading to hemoptysis.^3^, ^4^ In this case report, we present the challenging management of a second‐trimester pregnancy in an immunocompromised individual who experienced life‐threatening hemoptysis due to invasive pulmonary aspergillosis (IPA) accompanied by a co‐infection of pulmonary tuberculosis (PTB).

## CASE REPORT

A 37‐year‐old woman at 25 weeks of pregnancy presented with hemoptysis for 1 day. Her background medical history includes SLE in remission and PTB 10 years ago, where she completed anti‐tuberculous therapy for 6 months. Her SLE was stable on maintenance of cyclosporine 100 mg daily, hydroxychloroquine 200 mg daily and low‐dose prednisolone 5 mg daily. On presentation, she was afebrile with vital signs: blood pressure 112/72 mmHg, pulse rate 110 beats per minute, and saturation of 91% on room air.

**FIGURE 1:**
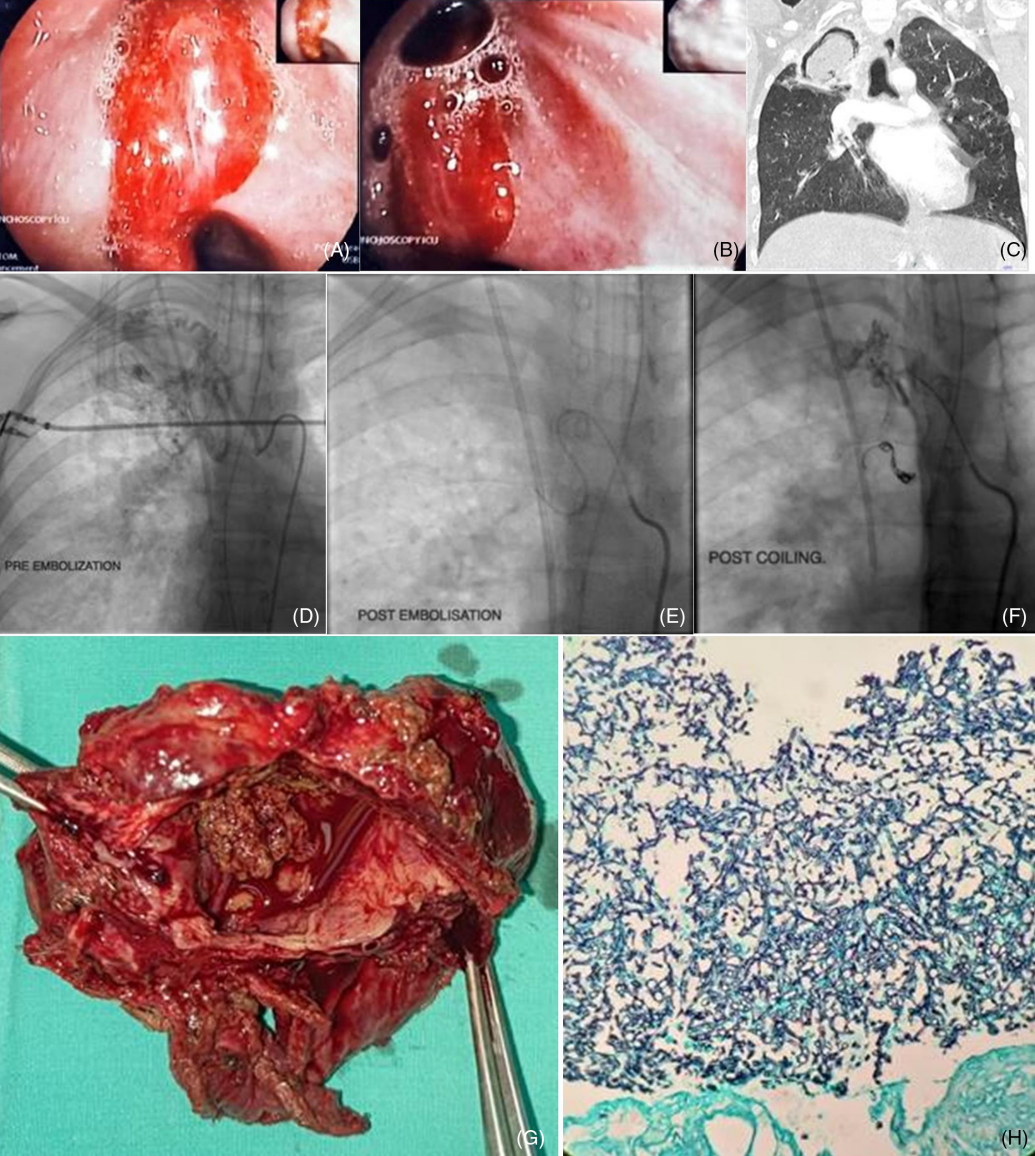
(A, B) Flexible bronchoscopy showed a clot originating from RB1 and RB 2 segments. (C) Chest CT (lung setting; coronal view) showed a large cavity with intra‐cavitary soft tissue mass at the right upper lobe with an air crescent sign. (D) The right bronchial artery angiogram shows multiple arteries supplying the right upper lobe cavity. (E, F) Post‐selective coil embolisation of right upper and lower bronchial arterial branches, thyrocervical artery, medial right internal mammary artery, and intercostal arteries supplying the cavity. (G) Resected specimens of the lung with dissected fibrotic cavity showing intraluminal mycetoma and blood clots. (H) Special GMS stain of lung tissue revealed numerous fungal bodies in the form of hyphae with septations and acute angle branching.

However, she developed massive hemoptysis in the emergency department, and she suffered a cardiorespiratory arrest requiring cardiopulmonary resuscitation for a total of 4 minutes. She was intubated. Bronchoscopy showed pooling of blood clots in both main bronchus and active blood oozing from the right upper lobe (RUL) apical segmental bronchus (RB1) and RUL posterior segmental bronchus (RB2) (Figure 1A,B) that was secured with adrenaline and cold saline flushing. An urgent CT has ruled out pulmonary embolism. However, it revealed an air crescent sign at the superior right upper lobe (Figure 1C), bilateral patchy peribronchovascular ground glass opacities with centrilobular densities, tree‐in‐bud patterns and multiple right bronchial artery dilatation encircling the cavity.

Following this, she was subjected to a successful image‐guided embolisation of the right bronchial artery and its branches supplying the cavitation lung mass with glue, lipiodol and microcoil (Figure 1D–F). Bronchoalveolar lavage fluid (BALF) showed positivity for mycobacterium tuberculosis (MTB) PCR (XPert MTB/RIF), negative for acid‐fast bacilli and was strongly positive for aspergillus antigen (ELISA) with an index of 7.29. The BALF MTB and fungal cultures yielded no growth.

We attributed her life‐threatening haemoptysis secondary to angioinvasive pulmonary aspergillosis. She was started on intravenous (IV) deoxycholate amphotericin B 0.7 mg/kg/day titrated to 1 mg/kg/day and an anti‐tuberculous regime of rifampicin (R), isoniazid (H), ethambutol (E), and pyrazinamide (Z). On day 3 of treatment, she developed isolated hyperbilirubinemia, which resolved after R substitution with moxifloxacin (M) 400 mg daily. Subsequently, we have decided on a combination antifungal of IV voriconazole 6 mg/kg two times a day for 1 day, followed by 4 mg/kg two times a day and IV anidulafungin 200 mg daily for 1 day, followed by 100 mg daily for one‐week duration, then monotherapy of oral voriconazole 4 mg/kg two times a day.

In the third week of therapy, she developed a second episode of pulmonary haemorrhage confirmed with bronchoscopy from RB 1 and RB 2; secured bronchoscopically with cold saline irrigation and adrenaline application, followed by bronchial artery embolization. Following this event, she underwent an emergency lower segment caesarian section at 28 weeks of pregnancy, after completion of intramuscular dexamethasone and IV magnesium sulphate for fetal lung maturation and neuroprotection, respectively. Four days later, she underwent video‐assisted thoracoscopic surgery (VATS) of the right upper lobectomy. Tissue was obtained intraoperatively, and histopathological examination showed hyphae with septations and acute angle branching, which confirmed the diagnosis of invasive pulmonary aspergillosis (Figure 1G,H). Throughout the hospitalization, she was maintained on low‐dose immunomodulatory IV hydrocortisone 50 mg twice daily.

She delivered a 920 g baby girl who was initially intubated for a low Apgar score and then successfully extubated the following day, and of note, no fetal malformation was observed. Subsequently, she could wean off ventilator support to room air saturation 1 week after the VATS. She has persistent air leak post‐operatively, managed conservatively with negative Sinapi® drain suction, with minimal residual non‐expandable lung.

She was discharged on day 19 post‐operative after the pulmonary air leak had resolved. Due to limited availability, the antifungal was changed to oral itraconazole 200 mg twice daily while continuing the HEZM anti‐tuberculous regime. Upon completion of antifungal therapy, HR were the drug of choice for the maintenance of the anti‐tuberculous regime. At the 6‐week follow‐up, she remained well with no recurrence of haemoptysis and improved radiological images.

## DISCUSSION

Our patient exhibited critical and life‐threatening hemoptysis due to a dual pathology confirmed by BALF and lung histopathological examination. Despite initial stability, she swiftly deteriorated, highlighting the significant impact of high‐pressure bleeding from the bronchial arteries. The bleeding's origin is probably attributed to vascular remodelling due to granuloma formation, leading to dysfunctional blood vessels and inhibition of angiogenesis, resulting in alveolar haemorrhage secondary to invasive aspergillosis and tuberculosis.^5^, ^6^ Additionally, physiological changes associated with pregnancy, such as increased blood volume and cardiac output, further contributed to the complexity of the case.^7^

Following securing the airway as per guidelines, an early bronchoscopy, guided by initial CT findings, was performed on our patient. The primary goal of this approach is to assess the airway for localization and control of bleeding, achieve endobronchial haemostasis, and enhance the diagnostic yield of BALF to identify concomitant infection. For our patient, despite a recurrence of hemoptysis, it was successfully managed through endobronchial and endovascular intervention.^8^, ^9^, ^10^ However, ongoing vigilance is crucial due to the expected nature of recurrence in the underlying disease. Surgical resection, which is timely decided, aligns with guidelines for recurrent hemoptysis due to invasive pulmonary aspergillosis and supports better obstetric outcomes and fetal growth. In our case, surgical intervention serves dual purposes: providing definitive treatment for recurrent hemoptysis and eliminating the cavity that poses future recurrence risks of aspergillosis, especially as the patient requires lifelong immunosuppressive therapy.^9^, ^11^, ^12^

Addressing the challenge of selecting antifungal and concomitant anti‐TB therapy during pregnancy is complex. Voriconazole, the first choice for IPA,^11^, ^12^, ^13^ poses fetal teratogenicity risks (category D), compounded by rifampicin co‐administration, reducing voriconazole serum concentration through CYP450 induction.^14^, ^15^ The absence of therapeutic drug monitoring complicates treatment efficacy and safety. In our case, amphotericin B was initially chosen, later transitioning to combination therapy with voriconazole post‐rifampicin removal following thorough counselling.

Although the Centers for Disease Control and Prevention do not recommend the use of pyrazinamide in pregnancy, it is extensively used in high‐burden countries. The choices of anti‐tuberculous are in line with WHO guidelines, including the British Thoracic Society, the American Thoracic Society, and the International Union Against Tuberculosis and Lung Diseases for the EHRZ regime as a first‐line therapy. It is not known to be harmful during pregnancy and can achieve better pregnancy outcomes. Studies have demonstrated that pyrazinamide, a key sterilizing agent, penetrates diseased pulmonary tissue well, including in cavitary‐type lesions.^16^, ^17^ Moxifloxacin was selected for its bactericidal properties and favourable safety profile. Case reports and prospective studies have reported a similar choice of fluoroquinolone as a rifampicin‐sparing regimen.^18^, ^19^, ^20^, ^21^ Navigating drug interactions and fetal risks underscores the intricacies of managing our patients' conditions.

Concurrent active PTB and IPA in immunocompromised pregnant individuals pose a high mortality risk. Swift control of bronchial artery bleed, employing a bronchoscopy approach and bronchial artery embolization, is crucial. Surgical lobectomy offers definitive therapy, especially given the long‐term need for immunosuppressants and recurrent hemoptysis. Managing the dual use of anti‐TB and antifungal medications presents therapeutic complexities, underscoring the importance of comprehensive assessment, vigilant monitoring, and prioritizing pregnancy safety.

## CONFLICT OF INTEREST STATEMENT

Dr Andrea Ban Yu‐Lin is an Editorial Board member of Respirology Case Reports and a co‐author of this article. She was excluded from all editorial decision‐making related to the acceptance of this article for publication. The other authors have no conflict of interest to declare.

## ETHICS STATEMENT

The authors declare that appropriate written informed consent was obtained for the publication of this manuscript and accompanying images.

## Data Availability

Data sharing not applicable‐no new data generated, or the article describes entirely theoretical research.
